# Spectrum of Ophthalmic Manifestations in Patients With Transfusion-Dependent Thalassemia

**DOI:** 10.7759/cureus.82218

**Published:** 2025-04-14

**Authors:** Pragya Jain, Sadaf Ikram, Yuri Kashiv, Madhu Chanchlani, Harpal Singh, Abhishek Singhai

**Affiliations:** 1 Ophthalmology, People's College of Medical Sciences and Research Centre, Bhopal, IND; 2 Pediatrics, People's College of Medical Sciences and Research Centre, Bhopal, IND; 3 Ophthalmology, Chirayu Medical College and Hospital, Bhopal, IND; 4 General Medicine, All India Institute of Medical Sciences, Bhopal, Bhopal, IND

**Keywords:** chelation therapy, ocular manifestations, refractive error, serum ferritin levels, transfusion-dependent thalassemia

## Abstract

Introduction: Thalassemia is a hereditary blood disorder characterized by impaired hemoglobin production, necessitating regular blood transfusions to manage anemia and associated complications. This condition also poses a significant risk for a range of ophthalmic manifestations due to factors such as iron overload from repeated transfusions, organ dysfunctions, and metabolic imbalances. This research aimed to evaluate the prevalence of ophthalmic anomalies in patients with transfusion-dependent thalassemia (TDT) and to ascertain their correlation with serum ferritin levels, hemoglobin concentrations, and the length of chelation therapy.

Materials and methods: This cross-sectional study was carried out at a tertiary care center in Central India in the pediatric and ophthalmology departments. All patients diagnosed with beta-thalassemia major between the ages of one and 15 were included in the study. Written informed consent was taken from the parents of participants. Patients with congenital ocular abnormalities, patients with a history of ocular trauma and surgery, and patients with hemoglobin diseases other than beta-thalassemia major were excluded. Complete medical history, including the disease's onset and course, blood transfusion frequency, splenectomy (performed or not), iron-chelating agents (nature, amount, time, and adherence to the regimen), positive consanguinity, and related conditions in the family, was recorded from the parents of every child. The ocular examination consisted of refraction, visual acuity, fundoscopy, slit-lamp examination, tonometry, perimetry in glaucoma suspects, tear break-up time (TBUT) test, and color vision testing. The data were analysed using the Statistical Product and Service Solutions (SPSS, version 19; IBM SPSS Statistics for Windows, Armonk, NY) software.

Results: Mean (±standard deviation) age of study participants was 8.10±3.83 years (age range: 1.5-14 years). Males comprised a smaller proportion of study participants than females, at 44% and 56%, respectively. We found one or more ocular manifestations in 38 (76%) of the patients, whereas the remaining 12 (24%) had none. Among all the ophthalmic manifestations, refractive errors were found in 56% of patients, followed by vascular tortuosity (32%). In this study, black pigmentation near the optic disc was found in 12% (6) of the patients, 4% had optic disc edema, and 4% had a high cup disc ratio. Yellowish pigmentation of the conjunctiva was seen in 8% of the patients, and 4% had dry eyes with TBUT shorter than 10 seconds.

Conclusion: Ophthalmic manifestations such as refractive errors, vascular tortuosity, pigmentation near the optic disc, and disc edema are common findings in thalassemia patients. This comprehensive study highlights the importance of regular ophthalmic evaluations in TDT patients, emphasizing the need for an interdisciplinary approach that includes hematologists and ophthalmologists. Early detection and management of ophthalmic complications can significantly enhance a patient's quality of life and preserve vision. Future research should focus on understanding the underlying mechanisms of these ocular manifestations and developing targeted interventions to mitigate their impact.

## Introduction

Thalassemia is the most prevalent single-gene condition in the world [1]. The hallmark of beta thalassemia is anomalies in the beta-globin chain production of hemoglobin, which can lead to a wide range of phenotypical results, from severe anemia to clinically asymptomatic persons. According to estimates, there are one in 100,000 new symptomatic cases of beta thalassemia, which are detected worldwide every year, and an estimated 10,000-15,000 babies are born with thalassemia each year in India [2]. There are about 17 million beta thalassemia carriers in India [2]. Growth retardation, delayed sexual maturity, hormonal problems (thyroid, parathyroid, and sex hormone deficits), diabetes, and cardiovascular problems are some of the systemic diseases related to thalassemia. Systemic illnesses related to thalassemia have several underlying causes, including iron overload, diminished somatomedin activity, multiple endocrinopathies, chronic hypoxia and anemia, low socioeconomic level, and racial or ethnic conditions [3]. Deferrioxamine and deferriprone are two regularly used iron-chelating medicines that help prevent systemic diseases resulting from siderosis. In addition to iron, these drugs cause other metals in the retina to chelate, including zinc, copper, cobalt, and nickel. The most necessary metals for the retina to function normally are nickel and cobalt, and deficiencies in these elements result in several ocular problems [3]. The disease may cause several undesirable eye changes, including cataract development, retinopathy, optic neuropathy, diminished visual acuity, altered color vision, and nyctalopia [3,4]. Studies on beta thalassemia have found that 41.3%-85% of cases may have eye involvement [4-9].

Numerous factors, including microvasculature illness, chronic anemia, iron overload and chelator toxicity, and aberrant orbit growth brought on by irregular craniofacial growth, can lead to ocular problems. Ocular surface diseases and lens opacities, which are reported in 9.3%-44% of cases, are common findings from many research studies about the eyes [6-10]. Desferrioxamine and deferiprone usage correlate positively with lenticular opacities and retinal pigment epithelium (RPE) degeneration. Ocular fundus abnormalities such as peau d'orange and angioid streaks are associated with pseudoxanthoma elasticum (PXE). Seven studies have found alterations resembling pattern dystrophy and drusen in the optic disc [6-10]. Retinal vascular tortuosity has been seen in 11%-17.9% of patients [9,10]. Apart from fundus examination, additional methods such as fundus autofluorescence and electrophysiological testing (electroretinogram and electrooculogram) might offer valuable information about the initial or more severe damage in the eyes of those affected by beta thalassemia [4].

This study sought to assess the occurrence of ocular anomalies in individuals with multi-transfusion beta thalassemia and to determine their relationship with serum ferritin levels, hemoglobin concentrations, and the duration of chelation therapy.

## Materials and methods

This cross-sectional study was conducted at People's College of Medical Sciences and Research Centre, Bhopal, India, in the pediatric and ophthalmology departments. All patients diagnosed with beta thalassemia major between the ages of one and 15 were included in the study. To keep their hemoglobin concentration between 9 and 11 g/dL, all the enrolled children have been getting treatment through packed red cell transfusion at a dose of 10 mL/kg body weight/transfusion. Iron-chelating drugs were started if the serum ferritin level was over 1,000 μg/dL. Written informed consent was taken from the parents of participants. This study was approved by the institutional ethical committee. Patients with congenital ocular abnormalities, patients with a history of ocular trauma and surgery, and patients with hemoglobin diseases other than beta thalassemia major were excluded. The sample size was calculated based on the prevalence of ophthalmic manifestations reported in previous studies and an estimated effect size of 0.2 with 80% power and a two-sided significance level of 0.05. Assuming a dropout rate of 10%, a total sample size of 50 participants was determined to be sufficient to detect clinically significant associations between thalassemia and ophthalmic complications.

Complete medical history, including the disease's onset and course, blood transfusion frequency, splenectomy (performed or not), iron-chelating agents (nature, amount, time, and adherence to the regimen), positive consanguinity, and related conditions in the family, was recorded from parents of every child. The pediatrician performed physical examination, including growth assessment, pallor, icterus, hepatosplenomegaly, frontal bossing, large maxilla, and skin hyperpigmentation at specific points. Hemoglobin concentration, serum ferritin, and complete blood count estimation were done in all patients.

Every patient received a comprehensive examination of their eyes by an ophthalmologist. The ocular examination included visual acuity assessment, refraction, fundoscopy, slit-lamp examination, tonometry, perimetry for glaucoma suspects, tear break-up time (TBUT) test, and color vision evaluation. An autorefractometer (mean of three measurements using an Autorefractor Keratometer Topcon KR-8900, Tokyo, Japan) was used to measure the refractive errors. Using a tumbling E chart with retro lighting with a brightness of 100 candela per square meter, monocular measurements of uncorrected and best-corrected visual acuity were made at a distance of 6 m. The procedures included ophthalmoscopy (Ophthalmoscope Heine Beta 200; HEINE Optotechnik, Germany), slit lamp biomicroscopy, and intraocular pressure monitoring with a noncontact Reichert seventh-generation noncontact tonometer. A TBUT test was done to detect dry eye condition. The TBUT was done under a slit light microscope with a cobalt-blue filter without the use of topical anaesthesia. The patient’s conjunctival sacs were filled with fluorescein solutions. To make sure the fluorescein was dispersed uniformly, the patients were told to blink many times for a few seconds. It was recorded how long it took for the first tear film to rupture after the last complete blink. It was considered dry eye if the TBUT was shorter than 10 seconds [10].

Glaucoma-suspect subjects underwent a 30-2 Swedish Interactive Thresholding Algorithm (SITA)-fast visual field (VF) examination using the Humphrey Field Analyser II (750 I Series; Carl Zeiss Meditec, Jena, Germany). The test duration was reduced by using the SITA-fast method. We consider each patient's total and pattern deviations, the global hemifield test, and the greyscale when interpreting VF results. Global indices were a way to quantify VF loss numerically. The data were analyzed using Statistical Product and Service Solutions (SPSS, version 19; IBM SPSS Statistics for Windows, Armonk, NY). Analysis of variance (ANOVA), Student's t-tests, Spearman correlation, Mann-Whitney U tests, and chi-square tests were used whenever needed. The type, dosage, and duration of chelation therapy, the number of blood transfusions received, and serum ferritin levels were correlated with the ocular characteristics of thalassaemia patients.

## Results

The study included a total of 50 children. Twenty-two (44%) of these were boys, while 28 (56%) were girls. The youngest was 1.5 years old, while the eldest was 14 years old (mean+SD age: 8.10±3.83). Table 1 shows hematological parameters of all study participants, including the requirement for blood transfusions; the average duration of chelation therapy is about seven years; and the dose varies substantially between patients, possibly due to their iron load and clinical condition. The average hemoglobin level is lower than normal, as is characteristic of thalassemia patients, underlining their reliance on transfusions. Serum ferritin levels are elevated, indicating significant iron overload in many cases.

**Table 1:** Distribution of hematological parameters among beta thalassaemic patients g/dL: gram/decilitre, ng/mL: nanogram/mililitre

Parameters	Minimum	Maximum	Mean	Standard Deviation
Total number of blood transfusions (n)	5	435	104.72	84.17
Duration of iron chelating therapy (years)	.00	13	6.8	3.83
Dose of chelating agent (mg)	.00	1250.00	571.00	273.83
Hemoglobin (g/dL)	4.5	9.50	7.38	1.35
Serum ferritin (ng/mL)	340.00	5967.00	1913.77	1375.73

After a thorough ocular examination, we found one or more ocular manifestations in 38 of the patients, whereas the remaining 12 had none (Table 2). Among all ocular manifestations, refractive errors were detected in 28 (56%) patients; however, all patients' refractive errors have been corrected using glasses, and all patients in this study had the best corrected visual acuity of 20/20. Vascular tortuosity in the retina was observed in 16 (32%) patients during a fundus examination; no special criteria or grading were employed in this study to define vascular tortuosity. A single senior consultant made the diagnosis based on clinical experience. In this study, black pigmentation near the optic disc was found in six (12%) patients. Two patients (4%) had optic disc edema, but there were no additional symptoms of increased intracranial tension. Two cases (4%) had a high cup disc ratio. Patients did not have high intraocular tension, and no glaucomatous alterations were identified during visual field tests. In this study, the mean intraocular pressure of the right eye was 15.18 mmHg (with SD of 2.933), while the mean intraocular pressure of the left eye was 14.4 mmHg. Yellowish pigmentation of the conjunctiva was seen in four patients (8%) during slit lamp examination. In this study, the mean TBUT of the right eye was 14.92 seconds (with SD of 3.029), whereas the mean TBUT of the left eye was 14.84 seconds (with SD of 3.222). Two (4%) patients were identified as having dry eyes with TBUT shorter than 10 seconds.

**Table 2:** Ocular manifestations of beta thalassemia patients

S. No.	Ocular manifestation	Number of patients	Percentage
1.	Refractive error	28	56
2.	Yellowish pigmentation of conjunctiva	4	8
3.	Dry eye	2	4
4.	Vascular tortuosity in retina	16	32
5.	Black pigmentation near optic disc	6	12
6.	Optic disc oedima	2	4
7.	Large cup disc ratio	2	4

On refraction testing, 22 (44%) of the patients were emmetropic, with normal vision of 20/20. Refractive error was present in 28 (56%) patients, with four (8%) being myopic and two (4%) being hypermetropic (Table 3). Myopic astigmatism was found in 12 (24%) patients, and hyperopic astigmatism in 10 (20%) patients, although each patient's best corrected visual acuity was 20/20. None of the patients was amblyopic.

**Table 3:** Distribution of refractive errors in study participants The p-value of 0.05 indicated statistical significance.

Refractive Errors	Frequency (n)	Percentage %	P-value
Emmetrope	22	44	0.005
Myopia	4	8	
Myopic Astigmatism	12	24	
Hyperopia	2	4	
Hyperopic Astigmatism	10	20	
Total	50	100	

In this study, ocular manifestations were seen in 38 (76%) of the patients, while only 12 (24%) did not have any. Patients with ocular manifestations received an average of 125.39 blood transfusions, compared to 39.25 for non-ocular manifestations, which was statistically significant (p<0.05). Individuals with ocular manifestation had a higher mean serum ferritin level (2183.03 ng/mL) compared to individuals without ocular manifestation (1,061.11 ng/mL), which was statistically significant (p<0.05) (Table 4). Patients with ocular manifestations had a longer period of chelating therapy (mean: 7.8 years) than patients with non-ocular manifestations (mean: 3.5 years). The difference was statistically significant (p<0.05). Patients with ocular manifestations received a higher dose of chelating agent and had a lower hemoglobin concentration than patients who did not have ocular manifestations, although the difference was not statistically significant.

**Table 4:** Comparision of variables with ocular manifestations in thalassemia patients g/dL: gram/decilitre, ng/mL: nanogram/mililitre

Parameters	Ocular manifestations in patients of thalassemia						
	NO (N=12)		YES (N=38)				
Variables	Mean	Standard deviation	Mean	Standard deviation	Mean Difference	T-test value	p-value
Numbers of blood transfusion (n)	39.25	35.33	125.39	84.74	-86.14	-3.40	0.001
Serum ferritin (ng/mL)	1061.11	552.06	2183.03	1451.06	-1121.92	-2.60	0.012
Duration of chelating therapy (years)	3.50	3.45	7.84	3.35	-4.34	-3.88	0.00
Chelating therapy dose (mg)	462.50	324.65	605.26	250.88	-142.76	-1.59	0.11
Hemoglobin (g/dL)	7.76	1.47	7.26	1.31	0.49	1.11	0.27

## Discussion

Thalassemia major, a hereditary disease, is more prevalent in the Mediterranean region, where consanguineous marriages are common [2]. The condition necessitates lifetime monitoring and treatment, posing a social and economic challenge. The life expectancy of thalassemia patients has grown as a result of modern therapeutic interventions. On the other hand, iron overload and continuous transfusion might result in numerous organ failures or dysfunction. To avoid iron accumulation in tissues, numerous iron-chelating compounds are utilized. Furthermore, thalassemia patients may experience unique adverse effects from iron-chelating therapy [11,12].

In this cross-sectional study, we looked at the prevalence and pattern of ocular manifestations in patients with transfusion-dependent thalassemia, as well as how they related to hematologic parameters. In our study, we selected 50 patients. Similar studies were conducted by Mondal et al. on 75 patients [13], Baig et al. on 203 patients [14], Haghpanah et al. on 79 patients [15], Jafari et al. on 54 patients [9], and Prasad et al. on 234 beta thalassemia major patients [16]. Our study's age range was 1.5-14 years, similar to that of Jafari et al. [9], who enrolled individuals aged six months to 21 years. Similarly, Abdel-Malak et al. [8] selected patients aged 6-16 years, Aksoy et al. [17] recruited patients aged 2-17 years, and Baig et al. [14] recruited 11-17-year-old children with beta thalassemia major for their study. Naseem et al. enrolled individuals aged 17-30 years [18], Haghpanah et al. recruited individuals aged 18-43 years [15], and Prasad et al. included individuals aged 25.6±6.3 years [16]. The last three studies included primarily adult patients.

Our study found that, out of 50 patients, 38 (76%) exhibited one or more ocular manifestations, with refractive error (56%) being the most common. Abdel-Malak et al. reported ocular manifestations in 85% of cases [8], and Baig et al. reported the same in 22.7% of cases [14]. In their study, refractive error was not considered an ocular finding. Gartaganis et al. [6] reported ocular findings in 41.3% of patients, while Jafari et al. reported in 68.5% of cases [9]. Abdel-Malak et al. found ocular manifestations in 58% of patients, with lower visual acuity primarily due to refractive error listed as an ocular finding [8]. According to our findings, 28 (56%) of our patients had decreased visual acuity due to refractive error, yet all of them had best-corrected visual acuity of 20/20. Abdel-Malak et al. also documented reduced visual acuity in 33% of their patients, with refractive error being the cause in all cases and totally correctable with glasses [8]. In contrast, Taher et al. [19] found impaired eyesight in thalassemic individuals at 19.4%, and Gosai et al. [20] found impaired visual acuity in 26% of their patients. In a study by Aksoy et al., visual acuity was reported to be lower than <20/20 in 23.2% of thalassemic patients [17]. Haghpanah et al. reported that only one patient had anisometropic amblyopia in the left eye. None of the patients had best-corrected visual acuity lower than 20/40 [15]. The best corrected visual acuity was 20/20 in all patients studied by Jafari et al. [9].

In our study, the leading cause of diminution of vision was refractive error, which was corrected with glasses; 56% of patients had refractive error, 8% had myopia, 4% had hypermetropia, 24% had myopic astigmatism, and 20% had hypermetropic astigmatism in both eyes. Baig et al. observed that the refractive error was found in only five (2.3%) patients [14]. Naseem et al. [18] found that refractive errors were present in 48.5%, 2% of which had hyperopic astigmatism, and 18% had myopic astigmatism. In his study, myopia was present in 18% of cases and hyperopia in 10.5% of cases [16]. Mondal et al. showed that, among 75 study subjects, 93.3% had refractive errors in either eye, 6.7% were emmetropic, 69.66% had hypermetropia, 38.66% had myopia, and 45.33% had astigmatism [13]. Prasad et al. reported that 20.5% of thalassemia patients had refractive errors, ranging from myopia to hypermetropia and astigmatism [16]. Jetani et al. showed that refractive errors were found in 14 cases (23%), such as myopia with astigmatism in 13 (21.7%) and only myopia in six subjects (10%) [21].

In our study, all patients' intraocular pressure was in the normal range, with a mean of 15.18±2.93 mmHg in the right eye and 14.44±2.92 mmHg in the left eye. Haghpanah et al. found that the mean intraocular pressure was 14.88±3.34 (6-25) mmHg [15]. In our study, the mean TBUT score was 14.92±3.02 seconds in the right eye and 14.84±3.22 seconds in the left eye; only 4% of cases had dry eye. Aksoy et al. found the mean TBUT score to be 9.62 ±1.28 seconds, which was normal [17]. Prasad et al. found that dry eye was present in 33.3% of cases [16], and the mean±SD TBUT among thalassemia patients was 14.92±3.02 seconds, significantly lower than that in the standard group (16.12±5.73 seconds, p=0.000).

In our study, vessel tortuosity was present in 32% of patients, while Abdel-Malak et al. found tortuous vessels in 11% of cases [8]. Haghpanah et al. found that vessel tortuosity was present only in 2.5% of their patients [15], and Prasad et al. noticed vascular tortuosity in 39.3% of cases [16]. In the study of Gosai et al., they found tortuous blood vessels only in 4.5% of cases [20]. Our study found optic disc hyperemia in 4% of patients, while Baig et al. found disc hyperemia in 1% of cases [15]. A large cup disc ratio was present in 4% of patients in our study. Haghpanah et al. found a high cup disc ratio in 3.8% of patients [15]. In a study by Taher et al., retinal pigment epithelium changes were present in 25% of patients [19]. In our study, we found black pigments near the optic disc only in 4% of patients. Our study also found optic disc edema in 4% of patients. In other studies, black pigment near the disc and optic disc edema are not common ocular manifestations.

In our study, increased occurrence of ocular changes was observed with increased serum ferritin and with a higher number of blood transfusions received, but without significant correlation with mean hemoglobin concentration. In the pediatric population, chelation with oral deferasirox was reported to be safe and effective [22]. In our study, the maximum patients were on oral deferasirox. No significant association was observed between ocular manifestations and the dose of chelating agents in our study. Baig et al. [14] also found that individuals with ocular manifestations had considerably higher median serum ferritin levels of 5,113 ng/mL (3140-6477) than those without, which were 3,857 (2,495-4,950). Abdel-Malak et al. found that higher serum ferritin and iron levels, as well as more blood transfusions, were associated with an increase in ocular abnormalities [8]. Jafari et al. found no significant link between ocular manifestations and mean serum ferritin level (p=0.627) or mean hemoglobin concentration (p=0.143). There was a statistically significant correlation (p=0.005) between the frequency of blood transfusions and the existence of ocular abnormalities [9]. Haghpanah et al. found no significant correlation between fundus abnormalities, visual acuity, or intraocular pressure and hematologic parameters (p>0.05) [15].

There are few limitations of our study. We only included pediatric patients in this study, and thus ocular manifestations that can emerge later in the disease's progression were excluded. Our study sample size (n=50) was small compared to other studies, and we did not include normal children of the same age as the control group.

## Conclusions

This comprehensive study highlights the importance of regular ophthalmic evaluations in transfusion-dependent thalassemia patients, emphasizing the need for an interdisciplinary approach that includes hematologists and ophthalmologists. Early detection and management of ophthalmic complications can significantly enhance a patient's quality of life and preserve vision. Future research should focus on understanding the underlying mechanisms of these ocular manifestations and developing targeted interventions to mitigate their impact.
